# Functionality of Silk Cocoon (*Bombyx mori* L.) Sericin Extracts Obtained through High-Temperature Hydrothermal Method

**DOI:** 10.3390/ma14185314

**Published:** 2021-09-15

**Authors:** Wei-Hsun Wang, Wen-Shin Lin, Chia-Hung Shih, Cheng-You Chen, Siao-Hong Kuo, Wei-Lin Li, Yung-Sheng Lin

**Affiliations:** 1 Department of Orthopaedics, Changhua Christian Hospital, Changhua 500209, Taiwan; cmch10011@gmail.com; 2 School of Medicine, Kaohsiung Medical University, Kaohsiung 807378, Taiwan; 3 Department of Golden-Ager Industry Management, Chaoyang University of Technology, Taichung 413310, Taiwan; 4 Department of Medical Imaging and Radiology, Shu-Zen Junior College of Medicine and Management, Kaohsiung 821004, Taiwan; 5 Department of Chemical Engineering, National United University, Miaoli 360001, Taiwan; sch@mdais.gov.tw (C.-H.S.); qaz86890123@gmail.com (S.-H.K.); vivian54625@gmail.com (W.-L.L.); 6 College of Medicine, National Chung Hsing University, Taichung 402202, Taiwan; 7 Department of Plant Industry, National Pingtung University of Science and Technology, Pingtung 912301, Taiwan; wslin@mail.npust.edu.tw; 8 Miaoli District Agricultural Research and Extension Station, Council of Agriculture, Miaoli 363201, Taiwan; 9 Ph.D. Program in Materials and Chemical Engineering, National United University, Miaoli 360001, Taiwan; wayne20410@gmail.com; 10 Institute of Food Safety and Health Risk Assessment, National Yang Ming Chiao Tung University, Taipei 112304, Taiwan

**Keywords:** cocoon, sericin, hydrothermal, extract, antioxidant, tyrosinase

## Abstract

Sericin, a textile waste, can be used for antioxidant and skin-whitening purposes. The hydrothermal method of extracting sericin is more eco-friendly than are chemical and enzymatic methods. In this study, silk cocoons were cut into pieces and then subjected to hydrothermal extraction at three temperatures (160, 200, and 220 °C) to obtain sericin extracts (Sericin160, Sericin200, and Sericin220, respectively). Antioxidant activity and tyrosinase inhibition were measured to determine the extracts’ effectiveness. Sericin220 was the strongest antioxidant, with total phenol content, total flavonoid content, and ferric reducing power of 62.19 ± 0.04 mg gallic acid equivalents/g dry weight, 0.07 ± 0.01 mg quercetin equivalent/g dry weight, and 181.49 ± 0.024 mg vitamin C equivalent/g dry weight, respectively. The half-maximal inhibitory concentrations for DPPH and ABTS free-radical scavenging ability were 6.41 ± 0.05 and 0.79 ± 0.37 mg/mL, respectively. Sericin220 also exhibited the highest tyrosinase inhibition activity (70.82 ± 4.1 mg vitamin C equivalent/g), indicating its whitening potential.

## 1. Introduction

Silk cocoons are made of a natural polymer protein material [1]. The primary component of a silk cocoon is fibroin, and the outside of a silk cocoon is covered by four layers of sericin with different molecular weights [2]. Sericin constitutes 25 to 30% of silk cocoons [2]. Degumming is usually necessary to source silk from cocoons in the textile industry [3]. The removal of sericin can improve the sheen, softness, smoothness, whiteness, and dyeability of fibers obtained from silk cocoons [4]. Therefore, the removed sericin solution is often an unused byproduct of the textile industry [5]. Approximately 50,000 tons of sericin worldwide are discarded every year, creating a burden on the environment [2]. Sericin contains 18 amino acids, among which the main ones include serine, histidine, glycine, threonine, tyrosine, aspartic acid, and glutamic acid [6]. In addition, sericin is a promising biological material because of its antioxidant capacity [7,8,9,10], moisturizing capacity [3], corrosion resistance [11], antibacterial activity [10,12], and protection against ultraviolet radiation [10,12].

Three types of methods are traditionally used to extract sericin from silk cocoons in the so-called degumming process, namely, chemical, enzymatic, and hydrothermal methods [13,14]. Chemical methods for sericin extraction involve the use of numerous chemicals, such as sodium bicarbonate, ammonia, organic solvents (tartaric and citric acid), and soap. These chemical-based methods are not environmentally friendly and may cause a high amount of organic charge [13]. Enzymatic methods are costly and are subject to more operational restrictions [14]. Hydrothermal methods have been used to produce several extracts, such as those from green coffee beans [15], macroalgae [16], wheat bran [17], *Lonicera flos* [18], *Himanthalia elongata* (brown seaweed) [19], *Cornus stolonifer* [20], and *Paulownia elongata* *x fortunei* [21]. In addition, the application of hydrothermal methods for sericin extraction is a feasible strategy.

Studies have examined hydrothermal methods for extracting sericin. Kumar et al. performed autoclaving treatment at 121 °C for 20 min to extract sericin from three Indian silkworm varieties [6]. One study discovered that increasing the duration of the hydrothermal extraction (to between 30 and 120 min) resulted in greater degradation of sericinoid proteins at 121 °C [22]. Another study indicated that the amino acid yield of sericin increases when the duration (10–60 min) and temperature (120–160 °C) of hydrothermal extraction are increased [23]. However, few studies have investigated hydrothermal processes for extracting sericin from silk cocoons at temperatures higher than 160 °C. Therefore, this study examines the effects of high temperatures (i.e., 160, 200, and 220 °C) on the properties of sericin extracts that have been obtained through the hydrothermal method.

## 2. Materials and Methods

### 2.1. Reagents

1,1-Diphenyl-2-picrylhydrazyl (DPPH), 2,2′-azino-bis (3-ethylbenzothiazoline-6-sulphonic acid) (ABTS), trichloroacetic acid, Trolox, iron (III) chloride, and L-dopa were obtained from Alfa Aesar (Tewksbury, MA, USA). Folin–Ciocalteu reagent, gallic acid, vitamin C, and mushroom tyrosinase were obtained from Sigma-Aldrich (St. Louis, MO, USA). Sodium carbonate was obtained from Riedel-de Haën (Seelze, Germany). Potassium ferricyanide, sodium hydrogen phosphate, and sodium dihydrogen phosphate were obtained from Showa Chemical (Tokyo, Japan). All the reagents were used as received, without further purification.

### 2.2. Material Preparation and Extraction

Silk cocoons (*Bombyx mori* L.) were collected from the Miaoli District Agricultural Research and Extension Station (Council of Agriculture, Executive Yuan) in Taiwan. First, silk cocoons were rinsed with deionized (DI) water and cut into small slices. Second, 2 g of sliced silk cocoons and 30 mL of DI water were placed in a Teflon cup and then into a high-temperature furnace. The heating program started at room temperature and was increased to one of the three set temperatures (160, 200, and 220 °C) at a heating rate of 5 °C/min; the set temperature was maintained for 1 h for extraction. The sericin extracts Sericin160, Sericin200, and Sericin220 (the numbers indicate their extraction temperatures) were collected after cooling to room temperature and dried by a freeze-drying procedure.

### 2.3. Determination of Antioxidant Capacity

#### 2.3.1. Total Phenolic Content

Measurement of the total phenolic content was conducted, per procedures established in previous studies [24,25]. Briefly, 200 μL of sericin extract was mixed with 200 μL of 0.5 M Folin–Ciocalteu reagent in a microcentrifuge tube and left to stand for 5 min. Thereafter, 200 μL of 10 *w/v* % Na_2_CO_3_ was added to the mixture and shaken with a vortex mixer for 1 min; subsequently, 400 μL of DI water was added and mixed evenly. The resulting mixture was kept for 1 h in the dark at room temperature and subsequently centrifuged at 3000 rpm for 10 min. Then, 200 μL of supernatant was pipetted into a 96-well plate, and absorbance was measured at 700 nm. The total phenolic content was expressed in milligrams of gallic acid equivalents per gram of dry weight of silk extract (mg GAE/g).

#### 2.3.2. Total Flavonoid Content

Measurement of the total flavonoid content was conducted, per procedures established in previous studies [26,27]. Briefly, 50 μL of sericin extract, 50 μL of 5% NaNO_2_, and 40 μL of CH_3_OH were dropped into a 96-well plate. The mixture was left to stand for 5 min; then, 10 μL of 10% AlCl_3_ was added to the mixture, which was allowed to stand for another 6 min. Next, 100 μL of 1 N NaOH was added, and the mixture was allowed to stand for 30 min. Absorbance was measured at 510 nm. The total flavonoid content was expressed in milligrams of quercetin equivalents per gram of dry weight of silk extract (mg QE/g).

#### 2.3.3. DPPH Free-Radical Scavenging Ability

DPPH free-radical scavenging ability was assessed, per the procedures established in previous studies [28]. First, 50 μL of sericin extract and 50 μL of 500 μM DPPH–ethanol solution were mixed together and kept in the dark for 30 min. Absorbance was measured at 517 nm. DPPH scavenging activity was calculated using the following equation:

$$\mathrm{DPPH}\;\mathrm{free}\;\mathrm{radical}\;\mathrm{scavenging}\;\mathrm{ability}\;\left(\%\right)=[1-(A_{517}\;of\;sample/A_{517}\;of\;blank)]\times 100\%.$$

The half-maximal inhibitory concentration (IC_50_) was considered to be the amount of sample required in order for DPPH radical scavenging ability to reach 50%. 

#### 2.3.4. ABTS Free-Radical Scavenging Ability

ABTS free-radical scavenging ability was assessed, per the procedures established in previous studies [25,29]. First, 250 μL of 7 mM ABTS and 250 μL of 2.45 mM potassium persulfate were mixed thoroughly and kept in the dark at 4 °C for 16 h. The background absorbance value of the liquid was controlled at approximately 0.7 ± 0.05, using ethanol. Next, 180 μL of the adjusted mixed solution and 20 μL of sericin extract were allowed to react for 10 min at room temperature in the dark, and absorbance was measured at 734 nm. ABTS scavenging activity was calculated using the following equation:

$$\mathrm{ABTS}\;\mathrm{free}\;\mathrm{radical}\;\mathrm{scavenging}\;\mathrm{ability}\left(\%\right)=[1-(A_{734}\;of\;sample/A_{734}\;of\;blank)]\times 100\%.$$

The IC_50_ was reported as the amount of sample required for ABTS radical scavenging activity to reach 50%. 

#### 2.3.5. Ferric Reducing Power

Reducing power was assessed, per the method established in previous studies [27,30]. First, 2 mM PBS buffer (pH 6.6), 100 μL of 1% K_3_Fe(CN)_6_, and 100 μL of sericin extract were mixed and allowed to react in a water bath at 50 °C for 20 min. The mixture was then removed from the water bath and cooled to room temperature, and 100 μL of 10% trichloroacetic acid was added to the mixture for 1 min. Next, 100 μL of supernatant was diluted with 100 μL of DI water, and 20 μL of 0.1% FeCl_3_ was added. After 10 min, the absorbance was measured at 700 nm. Vitamin C was used as the standard for determining the ferric reducing power, which was expressed as milligrams of vitamin C equivalents (VCE) per gram of dry weight of silk extract.

### 2.4. Tyrosinase Inhibition

Tyrosinase inhibition was assessed, per the method established in previous studies [31,32]. First, 80 μL of 5 mM L-dopa (dissolved in 67 mM phosphate buffer, pH 6.8) and 80 μL of sericin extract were dropped into a 96-well plate. Next, 40 μL of mushroom tyrosinase solution (2 units/reaction) was added to the mixture. After the mixture reacted for 30 min at 37 °C, its absorbance was measured at 475 nm. This experiment used vitamin C as the standard chemical. Tyrosinase inhibition activity was calculated using the following equation:

$$\mathrm{Tyrosinase}\;\mathrm{inhibition}\;\mathrm{activity}\;\left(\%\right)=[1-(A_{475}\;of\;sample/A_{475}\;of\;blank)]\times 100\%$$

### 2.5. Statistical Analysis

Each treatment was performed in triplicate, and the extracts’ properties were compared using SAS software (version 9.4, SAS Institute, Cary, NC, USA) through an analysis of variance. When a difference was significant (*p* < 0.05), the treatment means were compared through Fisher’s protected least significant difference test (LSD) test.

## 3. Results and Discussion

### 3.1. Extraction Yield

**Figure 1:**
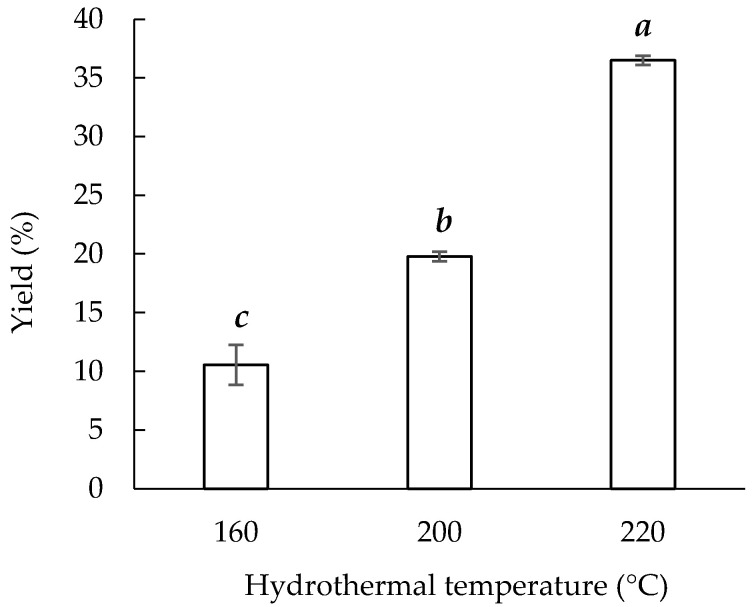
Hydrothermal extraction yields at various temperatures. Means with the same lowercase letters are not significantly different, per LSD test results.

The initial total sericin content was a protein solution. The extraction yield was defined as the weight ratio of the dried extract to the initial silk cocoons [13]. Figure 1 presents the extraction yields for Sericin160, Sericin200, and Sericin220, obtained hydrothermally at the corresponding temperatures. The results indicate that, among the three temperatures, the highest yield was achieved at 220 °C (36.50% ± 0.4%). At lower temperatures, the corresponding yield was lower; thus, the lowest yield was that for Sericin160 (10.54% ± 1.7%). The extraction yield of Sericin160 and Sericin200 were lower than the sericin content in the starting material, i.e., 25 to 30% of the silk cocoons [2]. One possible reason is the incomplete extraction of sericin from silk cocoons. A higher temperature provides more energy, to separate more sericin from silk cocoons, and higher solubility of sericin proteins to dissolve in the extraction solution [3]. Besides, a higher temperature may extract some fibroin to increase the extraction yield [23]. This finding corresponds to that of a previous work [23] that obtained larger sericin extraction yields through the hydrothermal method at higher temperatures (120–160 °C). In that study, the extraction yield also increased with the reaction time from 10 to 60 min.

### 3.2. Total Phenolic Content

**Figure 2:**
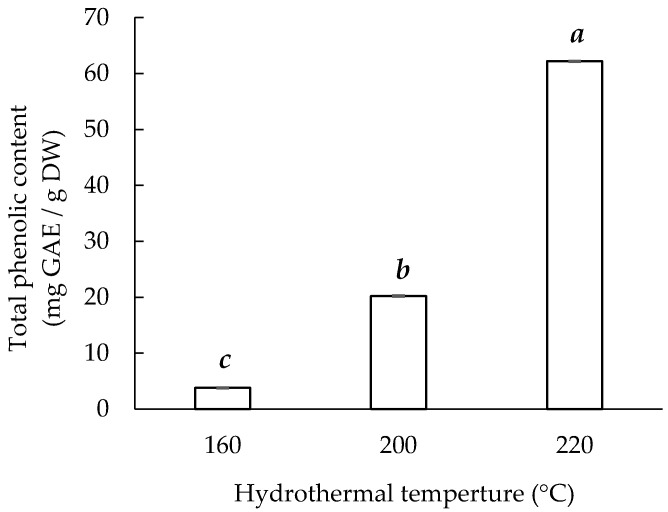
Hydrothermal extraction temperature and total phenolic content of sericin extracts. Means with the same lowercase letters are not significantly different, per LSD test results.

Studies have demonstrated phenols to be good antioxidants because of their strong antioxidant properties. Extracts with a high total phenolic content exhibit a high antioxidant capacity. Figure 2 indicates that Sericin220 had the highest total phenol content (62.19 ± 0.04 mg GAE/g) among the three sericin extracts, using the Folin–Ciocalteu method. The sericin extracts obtained at higher temperatures had a higher total phenolic content. Sericin220 had approximately twice the phenolic content of the other reported sericin extracts obtained through hydrothermal treatment (120 °C for 20 min), urea degradation, alkali degradation, and acid degradation [6]. Although the total phenol content via the Folin–Ciocalteu method, based on the electron transfer mechanism, may include reactions other than phenolic compounds [33,34], these results can still be regarded as reducing capacity for comparison between samples.

### 3.3. Total Flavonoid Content

**Figure 3:**
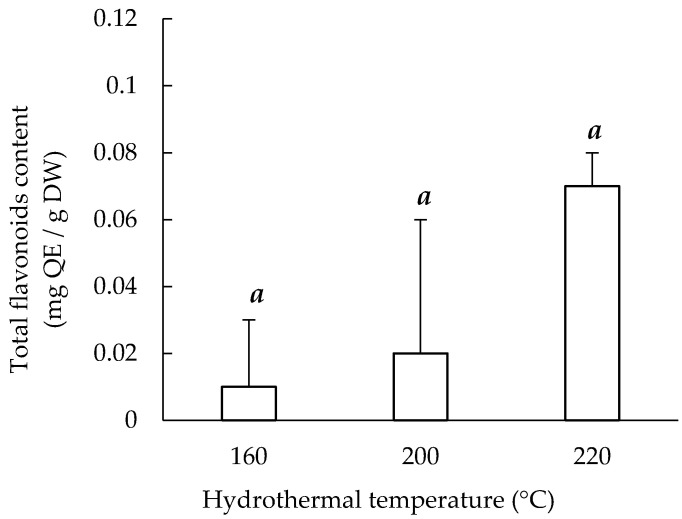
Hydrothermal extraction temperature and total flavonoid content of sericin extracts. Means with the same lowercase letters are not significantly different, per LSD test results.

The experimental results presented in Figure 3 indicate that Sericin220 had the highest total flavonoid content (0.07 ± 0.01 mg QE/g DW) among the three extracts, based on the assay of aluminum chloride complex formation. Overall, the total flavonoid content of the three sericin extracts was low, and no significant differences were observed among the three extracts because of large standard errors. One possible reason is the thermolabile property of sericin, with decomposition increasing according to reaction temperature and time [23]. Sericin will undergo significant hydrothermal degradation when in the vicinity of the boiling temperature of water [1]. The total flavonoid content of sericin extracts is related to the strain of *B. mori* silk cocoons used. A study tested the total flavonoid content of white, green, and yellow cocoons and discovered that green cocoons had the highest content levels [35]. However, the white cocoons that were treated using six solvents (i.e., acetone, ethyl acetate, dimethyl sulfoxide, ethanol, methanol, and purified water) at 25 and 50 °C for 4 h did not have any flavonoid content [35]. The flavonoid content of the white cocoons examined in the present study indicates that the method of extraction influences the flavonoid content of sericin extracts.

### 3.4. DPPH Radical Scavenging Ability

**Figure 4:**
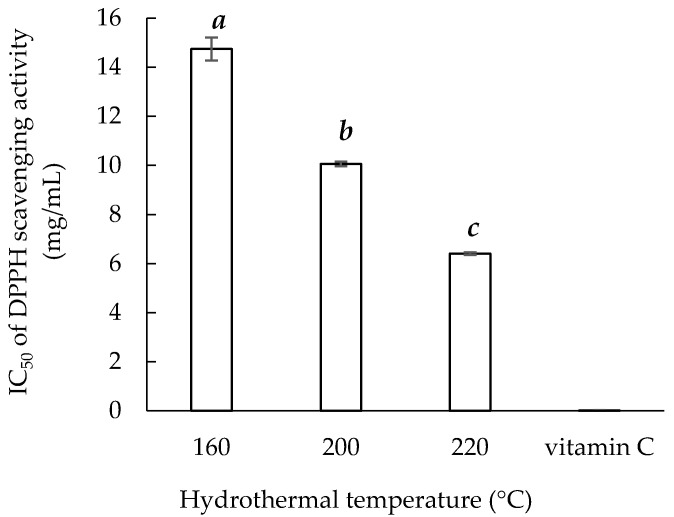
Hydrothermal extraction temperature and DPPH scavenging ability of sericin extracts. Means with the same lowercase letters are not significantly different, per LSD test results.

The IC_50_ of vitamin C for DPPH scavenging activity was 0.01 ± 0.02 mg/mL, and the IC_50_ of the sericin extracts obtained at the three temperatures are presented in Figure 4. Sericin220 had the lowest IC_50_ (6.41 ± 0.05 mg/mL). The IC_50_ values of Sericin160, Sericin200, and Sericin220 were lower than that reported in a previous study (IC_50_ = 31 mg/mL) for sericin extracted from silk wastewater with 75% (*v/v*) ethanol [36]. One possible reason is that the components of the sericin extracts were affected by the extraction process. The molecular size of sericin is significant for DPPH scavenging activity, and sericin extracted using the hydrothermal method had a lower IC_50_ than sericin extracted using a Na_2_CO_3_ solution [13].

### 3.5. ABTS Radical Scavenging Activity

**Figure 5:**
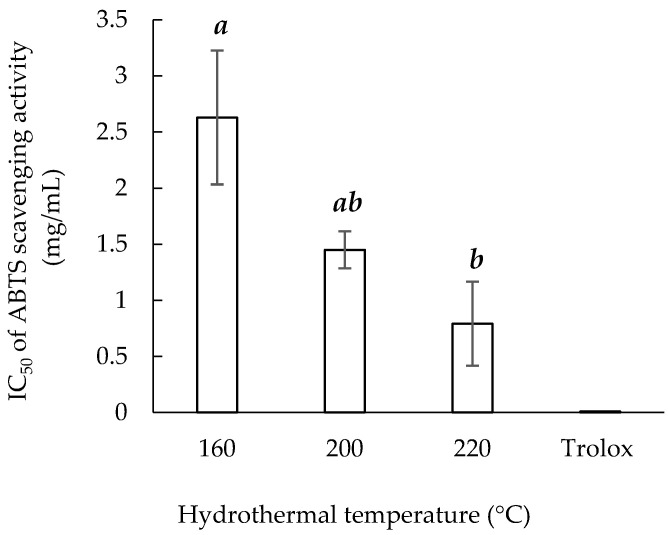
Hydrothermal extraction temperature and ABTS scavenging ability of sericin extracts. Means with the same lowercase letters are not significantly different, per LSD test results.

Figure 5 presents the IC_50_ values of Trolox and the three sericin extracts for ABTS scavenging activity. The low experimental IC_50_ value of Trolox was used to validate this experiment [37]. Among three extracts, Sericin220 (0.79 ± 0.37 mg/mL) had the highest ABTS radical scavenging ability, that is, the lowest IC_50_. Compared with those for DPPH radical scavenging ability, the IC_50_ values for ABTS radical scavenging ability were lower. The different solubility of the DPPH (oil soluble) and ABTS (oil- and water-soluble) radicals may explain why the extracts had greater ABTS radical scavenging ability than DPPH radical scavenging ability [38].

### 3.6. Ferric Reducing Power

**Figure 6:**
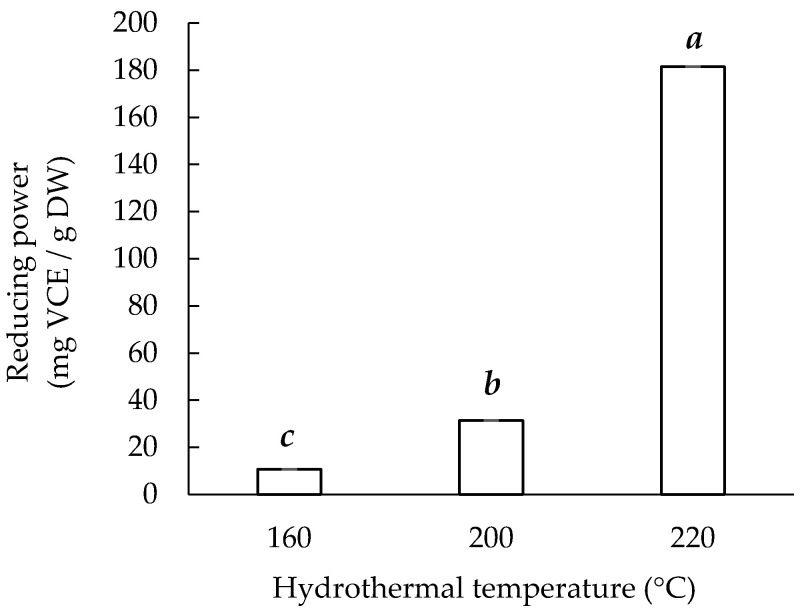
Hydrothermal extraction temperature and ferric reducing power of sericin extracts. Means with the same lowercase letters are not significantly different, per LSD test results.

Figure 6 presents the ferric reducing power of the sericin extracts obtained hydrothermally at three temperatures. Sericin220 had the highest reducing power (181.49 ± 0.02 mg VCE/g), followed by Sericin200 (31.36 ± 0.05 mg VCE/g) and Sericin160 (10.58 ± 0.01 mg VCE/g). These findings are supported by the total phenol content and antioxidant activity (DPPH and ABTS radical scavenging) results.

### 3.7. Tyrosinase Inhibition Activity

**Figure 7:**
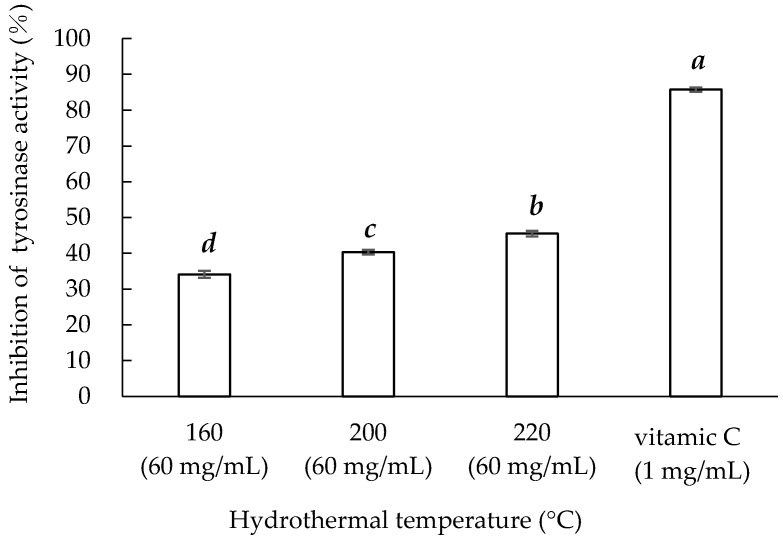
Hydrothermal extraction temperature and tyrosinase inhibition activity of sericin extracts. Means with the same lowercase letters are not significantly different, per LSD test results.

Most commercial cosmetics and skin-lightening agents contain tyrosinase inhibitors [39]. Figure 7 presents the tyrosinase inhibition activity of the three sericin extracts and standard vitamin C. Vitamin C at 1 mg/mL still exhibited greater inhibitory activity than the three sericin extracts at 60 mg/mL. Among the sericin extracts, Sericin220 exhibited the highest tyrosinase inhibition activity at 70.824 ± 4.1 mg VCE/g.

The property of the obtained sericin varies according to the extraction method. The molecular weight of sericin can vary from 5 kDa to 400 kDa [3]. Compared to chemical extraction, water extraction can maintain the primary structure and molecular weight of sericin [13]. The sericin amino acid composition can be determined by high-performance liquid chromatography. The amino acid composition of sericin includes serine, aspartic acid, glutamic acid, glycine, histidine, arginine, threonine, alanine, proline, cysteine, tyrosine, valine, methionine, lysine, isoleucine, leucine, phenylalanine, and tryptophan [3,36]. Among these amino acids, serine, aspartic acid, glycine, glutamic acid, and threonine occupy the significant molar percentage (> 70%) in all the reviewed extraction conditions [3], and serine and aspartic acid are in the majority [12]. Sericin contains a large amount (> 60%) of polar amino acids with functional groups, such as hydroxyl, carboxyl, and amino groups [13,40]. The molar percentage of serine and threonine having hydroxyl groups is greater than 30% [3]. Therefore, these functional amino acids may bring about the antioxidant and tyrosinase inhibitory activity of sericin [36]. For example, hydrophobic amino acids (e.g., glycine, alanine, valine, proline, leucine, and phenylalanine), with a molar amount greater than 20% [3], favor peptide interaction with lipids and enhance radical scavenging activity [41]. In particular, tyrosine and phenylalanine make a large contribution to radical scavenging because the indole and benzene ring can donate robust protons for reaction with electron-deficient radicals [42]. Besides, arginine and valine with a molar amount greater than 5% possibly account for tyrosinase inhibitory activity, due to tyrosinase binding and tyrosinase inhibition [3].

## 4. Conclusions

Silk cocoons play a key role in the textile industry. However, the sericin in silk cocoons is traditionally treated as waste. Sericin contains many functional ingredients, and its application in skin care products is attracting increasing attention. In the present study, high temperatures (160, 200, and 220 °C) were used to extract sericin hydrothermally. The results indicate that hydrothermal treatment at 220 °C is a favorable strategy for extracting sericin with a high yield, high antioxidant ability, and strong tyrosinase inhibition. The chemical-free hydrothermal extraction of sericin has less toxicity and better bioavailability in cosmetic applications. However, energy expenditure should be investigated in further work regarding its practical use in industries.

## Data Availability

All the data is available within the manuscript.
